# In Situ Loading and Time‐Resolved Synchrotron‐Based Phase Contrast Tomography for the Mechanical Investigation of Connective Knee Tissues: A Proof‐of‐Concept Study

**DOI:** 10.1002/advs.202308811

**Published:** 2024-03-23

**Authors:** Hector Dejea, Maria Pierantoni, Gustavo A. Orozco, E. Tobias B. Wrammerfors, Stefan J. Gstöhl, Christian M. Schlepütz, Hanna Isaksson

**Affiliations:** ^1^ Department of Biomedical Engineering Lund University Box 118 Lund 221 00 Sweden; ^2^ MAX IV Laboratory Lund University Lund 224 84 Sweden; ^3^ Swiss Light Source Paul Scherrer Institute Villigen PSI 5232 Switzerland

**Keywords:** articular cartilage, biomechanics, image quality, meniscus, phase contrast imaging, radiation damage, rheometer

## Abstract

Articular cartilage and meniscus transfer and distribute mechanical loads in the knee joint. Degeneration of these connective tissues occurs during the progression of knee osteoarthritis, which affects their composition, microstructure, and mechanical properties. A deeper understanding of disease progression can be obtained by studying them simultaneously. Time‐resolved synchrotron‐based X‐ray phase‐contrast tomography (SR‐PhC‐µCT) allows to capture the tissue dynamics. This proof‐of‐concept study presents a rheometer setup for simultaneous in situ unconfined compression and SR‐PhC‐µCT of connective knee tissues. The microstructural response of bovine cartilage (*n* = 16) and meniscus (*n* = 4) samples under axial continuously increased strain, or two steps of 15% strain (stress–relaxation) is studied. The chondrocyte distribution in cartilage and the collagen fiber orientation in the meniscus are assessed. Variations in chondrocyte density reveal an increase in the top 40% of the sample during loading, compared to the lower half. Meniscus collagen fibers reorient perpendicular to the loading direction during compression and partially redisperse during relaxation. Radiation damage, image repeatability, and image quality assessments show little to no effects on the results. In conclusion, this approach is highly promising for future studies of human knee tissues to understand their microstructure, mechanical response, and progression in degenerative diseases.

## Graphical abstract

In this study, time‐resolved synchrotron‐based X‐ray phase contrast tomography is used with concurrent unconfined compression to capture microstructural dynamics of bovine meniscus and articular cartilage. The study illustrates how collagen fibers and chondrocyte density alter during compression. The results show the potential of the presented approach for future studies of microstructure and mechanical response of human knee tissues during degeneration.

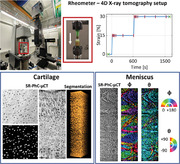

## 1 Introduction

Articular cartilage and meniscus are connective tissues that transfer and distribute mechanical load in the knee joint. Degeneration of both these tissues occurs during the progression of knee osteoarthritis, which alters the tissues’ composition and microstructure, and subsequently reduces their mechanical competence.^[^ ^1^, ^2^, ^3^ ^]^ However, the interrelationship between microstructure and mechanical response remains challenging to resolve.

Articular cartilage is mainly composed of water and a matrix of collagen type II and proteoglycans, which are maintained by chondrocytes.^[^ ^4^ ^]^ Both tissue composition and structure drastically change through the depth of the tissue. In the deeper zone, collagen is aligned perpendicular to the subchondral bone and the concentration of proteoglycans is highest, while toward the superficial layer, the collagen tends to become parallel to the surface and the proteoglycan content is the lowest. Chondrocytes, surrounded by a peri‐cellular matrix, are responsible for the preservation of healthy articular cartilage.^[^ ^5^, ^6^, ^7^, ^8^ ^]^ In the superficial zone chondrocytes have a flattened shape, which becomes rounder and wider in the middle zone. In the deep zone, chondrocytes are organized in vertical columns.^[^ ^1^, ^9^ ^]^ The meniscus consists of hierarchically arranged collagen type I fibers with depth‐dependent orientation.^[^ ^10^ ^]^ At the surface, the collagen fibers are arranged in a mesh‐like woven matrix.^[^ ^11^ ^]^ Under the surface, fibers form bundles and orient circumferentially around the meniscus.^[^ ^12^, ^13^ ^]^ The layered organization and anisotropic orientation of the fibers contribute to determining the mechanical response so that vertical compressive and shear loads can be effectively transferred and redistributed within the layers.^[^ ^14^, ^15^, ^16^ ^]^ Degeneration in both types of tissue is related to the progression of knee osteoarthritis (OA), which is presented as changes in the tissue microstructure and its mechanical properties, such as depletion of proteoglycans, weakening and disorganization of the collagen network, increased calcification and reduced chondrocyte density, all resulting in abnormal load distribution and high local peak stresses.^[^ ^17^ ^]^

Imaging techniques enable the structural characterization of tissues, such as the connective knee tissues. Magnetic resonance imaging provides good soft tissue contrast in three dimensions (3D) but is limited in spatial (≈1 mm) and temporal resolution, limiting the ability of microstructural assessment.^[^ ^18^, ^19^, ^20^, ^21^, ^22^, ^23^ ^]^ Laboratory‐based micro‐computed tomography (lab‐µCT) can achieve 3D micrometer‐level spatial resolution. However, scan times are long, and soft tissue contrast remains limited unless dedicated fixation^[^ ^24^, ^25^ ^]^ or contrast agents^[^ ^26^ ^]^ are used. Histological techniques are the gold standard for microstructural characterization due to the high spatial resolution (<1 µm) and the possibility of using staining, which dramatically enhances the contrast between structures of interest.^[^ ^11^ ^]^ However, tissue processing and sectioning can affect the tissue such that the 3D interpretation of sectioned samples can be misleading. Many of these limitations can be overcome by using synchrotron radiation‐based X‐ray phase contrast µCT (SR‐PhC‐µCT), as it combines 3D high resolution at high acquisition speeds and with little to no sample processing, albeit with a trade‐off between desired spatial and temporal resolutions.

SR‐PhC‐µCT has recently gained attention for pre‐clinical imaging in 3D) with high spatial resolution. As a result of the partial coherence of synchrotron X‐rays, SR‐PhC‐µCT is sensitive to differences in refractive indices between tissue types. For soft tissues, such differences can be several orders of magnitude higher than in conventional absorption.^[^ ^27^ ^]^ This technique has previously been used to characterize the microstructure of rodent,^[^ ^28^, ^29^ ^]^ bovine,^[^ ^30^ ^]^ and human cartilage,^[^ ^18^, ^31^, ^32^, ^33^, ^34^ ^]^ as well as human meniscus,^[^ ^35^ ^]^ allowing to differentiate cellular and collagenous fiber structures, and showing some potential to distinguish some signs of OA‐related degeneration. However, these studies have focused on static measurements, without considering the tissues’ mechanical and microstructural responses under loading conditions.

Another major advantage of SR‐PhC‐µCT is that the fast imaging times enable time‐resolved tomography (four dimensions, 4D) to capture the dynamics of musculoskeletal tissues through so called in situ mechanical testing.^[^ ^36^, ^37^, ^38^, ^39^ ^]^ This approach allows us to visualize in real‐time how the microstructure responds to the applied load, thus providing an enhanced picture of the relation between mechanical properties and structure at different length scales. With regard to connective knee tissues, this has previously been applied to study deformation in whole mouse joints^[^ ^37^ ^]^ and porcine cartilage samples^[^ ^40^ ^]^ loaded in situ. Nevertheless, imaging was only performed in fully relaxed states before and after loading, thus becoming insensitive to the dynamic mechanical and microstructural response and the relaxation process. No in situ studies on meniscal tissues are available to the best of our knowledge.

X‐ray diffraction or scattering techniques have also been widely used to study collagen orientation and strain in situ,^[^ ^41^, ^42^, ^43^, ^44^ ^]^ with the great advantage of being sensitive to tissue nanostructure. However, these often rely on two‐dimensional (2D) scanning measurements that average the results across the sample thickness (thus losing 3D spatial organization) and are not sensitive to non‐diffracting/scattering structures. Tensor tomography allows the expansion of measurements to the 3rd dimension,^[^ ^45^, ^46^, ^47^, ^48^, ^49^ ^]^ but it considerably increases acquisition time so that in situ experiments become challenging.

This proof‐of‐concept study presents the use of a customized rheometer setup for in situ loading with synchrotron radiation‐based phase contrast imaging of connective knee tissues. This approach allows to acquire full tomographic volumes at sufficient spatial and temporal resolution to study features of interest while a range of loading protocols are being applied. The aim is first of all to illustrate the possibilities offered by this methodology, and second to quantify the dynamic microstructural changes in fresh‐frozen bovine knee articular cartilage and meniscal samples under different mechanical loading protocols.

## 2 Experimental Section

### 2.1 Sample Description

**Figure 1:**
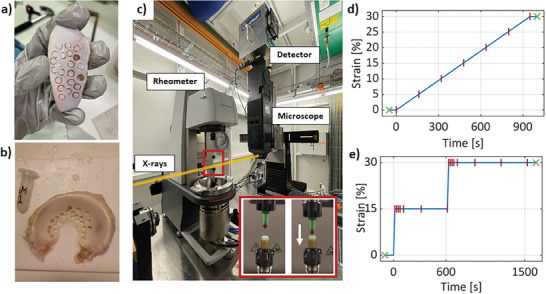
Methodological summary. a) Pictures of bovine articular cartilage and b) meniscus with representative sample extraction. c) Imaging setup at the TOMCAT beamline, consisting of a rheometer and TOMCAT's time‐resolved tomography setup. Includes zoom‐in of the sample holder geometry with an articular cartilage sample immersed in PBS. d,e) Illustrative continuous loading and stress‐relaxation protocol, respectively, with static (green x) and dynamic (red |) scanning times indicated.

Healthy articular cartilage (AC) and medial meniscal (MM) tissue samples (4 mm diameter) were harvested from a 4‐month‐old bovine knee joint obtained from the local slaughterhouse. AC and MM tissue samples were collected from the femoropatellar groove and MM, respectively (**Figure** **1**a,b). After extraction, the samples were stored in phosphate‐buffered saline (PBS) solution (0.15 m) and frozen at −22 °C until the day of use. On the day of the experiment, each individual sample was thawed to room temperature by immersion in PBS solution ≈10 min before imaging.

For this proof‐of‐concept study, a total of 16 AC and 4 MM tissue samples were investigated. From these, 2 AC and 2 MM samples were used for in situ loading, 1 AC and 1 MM samples were used for image quality analysis, 1 AC and 1 MM samples were used for repeatability analysis, and 12 AC samples were used for radiation damage analysis (Table S1, Supporting Information).

### 2.2 In Situ Loading and Time‐Resolved Synchrotron‐Based Phase‐Contrast Computed Tomography

#### 2.2.1 Experimental Setup

The synchrotron‐based tomographic experiments were performed at the TOMCAT beamline (Swiss Light Source, Paul Scherrer Institute, Switzerland).^[^ ^50^ ^]^ Time‐resolved phase contrast imaging of mechanically loaded tissue samples was achieved using TOMCAT's time‐resolved tomography setup in combination with a rheometer setup (Anton Paar MCR 702e MultiDrive). Custom 3D‐printed sample holders (5.5 mm inner diameter cups) and indenters (5 mm diameter) of photopolymerizable resin were used to apply in situ loading (Figure 1c–e).

Imaging was conducted with a monochromatic X‐ray energy of 21 keV and 40 cm sample‐detector distance. X‐rays were converted to visible light by a LuAG:Ce 150 µm scintillator, magnified by a fourfold high‐numerical aperture macroscope,^[^ ^51^ ^]^ and recorded by the GigaFRoST detector.^[^ ^52^ ^]^ This configuration resulted in projections with a pixel size of 2.75 µm and a field of view of 2016 × 1400 pixels (5.54 × 3.85 mm^2^). A detailed summary of acquisition parameters can be found in Table S2 (Supporting Information).

A PandABox^[^ ^53^ ^]^ was used to synchronize the mechanical loading and the continuous rotation of the rheometer with image data acquisition at desired time intervals, as explained below.

#### 2.2.2 Image Acquisition and Reconstruction

Samples were thawed and mounted in a hydrated state (PBS) on the custom holders. Initially, a series of scan times (40, 20, 10, 5, and 1 s – see Table S3, Supporting Information) were tested to find the most suitable acquisition parameters. After visually inspecting the reconstructions, 5 s scans were identified as the fastest acquisition that provided enough image quality for processing of dynamic data.

So‐called *static scans* (40 s scan, 9 ms exposure time, 4000 projections) were recorded before and after each mechanical protocol, as they provided the best image quality. During in situ loading, so‐called *dynamic scans* (5 s scan, 2.5 ms exposure time, 2000 projections) were taken at specific time intervals, depending on the applied protocol. All scans were acquired over 180°.

Additionally, 400 flats and 50 darks were acquired either before or after each sample was studied. This is required to correct the data for X‐ray beam inhomogeneities and detector noise. Flat‐ and dark‐field correction was achieved using dynamic intensity normalization,^[^ ^54^ ^]^ while the Gridrec algorithm^[^ ^55^ ^]^ in combination with the Paganin single‐distance phase retrieval algorithm^[^ ^56^ ^]^ was used to reconstruct the phase‐enhanced tomographic scans. Paganin was applied with δ/β ratio = 67, where delta and beta are related to the phase and absorption properties of the studied material, respectively, as defined by the index of refraction *n*  =  1 − δ + *i*β.

#### 2.2.3 Mechanical Protocols

To illustrate the potential of this setup for the study of knee connective tissue, AC and MM samples were subjected to unconfined axial compression in either continuous loading (CL) or stress–relaxation (StR). In all experimental protocols, samples were placed between two impermeable plastic plates of the custom 3D‐printed cup, and a pre‐load of 2 N was applied to ensure proper contact between the loading plate and the sample. Prior to the mechanical tests, each sample thickness was measured using the corresponding static scan reconstruction.

- Unconfined compression continuous loading: After pre‐load, a constant strain rate of 0.03% strain/s was applied continuously until either 15% (AC) or 30% strain (MM) was achieved. During this time, a total of seven dynamic scans were equidistantly acquired along the protocol.
- Unconfined compression stress–relaxation: After pre‐load, two compression stress–relaxation steps were applied (15% strain each step, speed 1% strain/s, followed by 600 and 900 s relaxation for the first and second steps, respectively). During the loading protocol, 15 dynamic scans were acquired at the compression peaks and at relaxation times of 20, 40, 60, 100, 300, and 600 s. In the following, relaxation times for the first and second steps are referred to as t_1_ and t_2_, respectively.

Loading speeds and imaging time intervals were chosen to minimize motion artefacts while keeping the 5 s dynamic scan and asses the dynamic processes undergone by the samples.

### 2.3 Image Processing

#### 2.3.1 Image Quality Assessment

Image quality was assessed by means of spatial resolution, signal‐to‐noise ratio (SNR), and contrast‐to‐noise ratio (CNR), according to previous 3D and in situ phase contrast studies in the literature.^[^ ^57^, ^58^, ^59^, ^60^ ^]^ Spatial resolution was calculated following a Fourier analysis criterion,^[^ ^61^ ^]^ in which the image's mean power spectral density is obtained. The power spectral density converges to the noise baseline at a specific frequency, which can then be converted to spatial resolution. SNR was defined as the ratio of average foreground intensity (AC matrix or MM collagen fibers) to the standard deviation of the background. CNR between image features “A” and “B” was defined as

(1) $$CNR_{A,B}=\frac{I_{A}-I_{B}}{\sqrt{\frac{1}{2}\left(\sigma_{A}^{2}+\sigma_{B}^{2}\right)}}$$

where I and σ correspond to the average image intensity and standard deviation for a specific feature, respectively.^[^ ^62^ ^]^ For AC, CNR was calculated between the extracellular matrix and chondrocytes, and between sample and background. CNR was calculated in MM between collagen fibers and extracellular matrix, and between sample and background.

#### 2.3.2 Articular Cartilage Analysis

For image analysis, 5 full‐depth sub‐volumes of 500 × 500 µm^2^ were cropped from each tomographic reconstruction with the goal of reducing computational cost and avoiding image artefacts, while still achieving a sufficient representation of the sample microstructure.

Chondrocytes were segmented using the Pixel Classification module in the software Ilastik.^[^ ^63^ ^]^ Using custom MATLAB scripts, the AC datasets were divided into 10 equally thick layers across the sample's height. Based on the segmentation, chondrocyte density was computed as percentual volume within the sample, as the image contrast was not sufficient to differentiate single chondrocytes when they were organized in columns.

#### 2.3.3 Meniscus Analysis

For image analysis, 5 sub‐volumes of the full meniscus depth × 500 × 500 µm^3^ were cropped from each tomographic reconstruction for collagen fiber orientation analysis. The orientation was computed using the structure gradient tensor method,^[^ ^64^, ^65^ ^]^ as previously applied to static imaging of human meniscus tissue^[^ ^35^ ^]^ and rat Achilles tendons.^[^ ^44^ ^]^ Briefly, images were initially smoothed using a Gaussian filter (standard deviation of 1). A tensor containing the image intensity gradient in 3D was then computed for every pixel in the image using a Sobel filter. The tensors were then further smoothed with a Gaussian filter (standard deviation of 4). Smoothing levels were chosen to reduce noise while maintaining the fiber orientation. Principal component analysis was used to obtain the three main eigenvalues and corresponding eigenvectors. In this case, the third eigenvector corresponds to the direction of the collagen fibers since image intensity will change the least along the fibers and the most perpendicularly across them. The orientation is then calculated in spherical coordinates and expressed as azimuthal (ϕ, [0 180] degrees) and elevation angles (θ, [−90 90]°), which describe the orientation on the cross‐sectional and longitudinal planes, respectively (Figure S1, Supporting Information). Azimuth and elevation angle histograms were calculated and compared across the levels of mechanical loading.

#### 2.3.4 Repeatability Analysis

To assess the differences arising from measuring the same sample in two different instances, 1 AC and 1 MM tissue samples were scanned at both 5 and 40 s scan times, removed from the rheometer, placed back, and re‐scanned with the exact same parameters. After rigid registration of the corresponding scans in MATLAB, the analysis steps explained in Sections 2.3.2 and 2.3.3 were applied to matching subvolumes of size 0.825 × 0.825 × 0.825 mm^3^ (300 × 300 × 300 pixels) for AC and size 0.825 × 0.825 × 3.4 mm^3^ (300 × 300 × 1236 pixels) for MM. Chondrocyte density and collagen fiber orientation were compared between the two repeated scans for each of the two different scan times.

### 2.4 Mechanical Analysis

The recorded force‐displacement data were used to calculate the mechanical properties of the samples. The data was converted to stress–strain curves by assuming a constant sample diameter of 4 mm across all samples, and the height measured from the tomography data. For both CL and StR protocols, the instantaneous Young's modulus (*E*_i_) was calculated from the slope of the stress–strain curve at each corresponding load step (15% or 30% strain). For StR only, the quasi‐equilibrium Young's modulus (*E*_qeq_) was calculated as the slope between the full‐relaxation stress at 15% and 30% strain. This value is referred to as quasi‐equilibrium as previous literature indicated that up to 22 min could be necessary for full MM relaxation.^[^ ^66^ ^]^ Further, the relaxation ratios were calculated as the ratio between the equilibrium and peak stresses at 15% and 30% strain. The equilibrium Poisson's ratio (ν_s_) was calculated by measuring the lateral strains to the corresponding axial strains (15% and 30%) on both AC and MM samples using the tomographic images from StR. The measurements were carried out using ImageJ.^[^ ^67^ ^]^ The aggregate modulus (H_A_) was calculated indirectly using Equation (2), which relates the equilibrium Young's modulus and the measured average Poisson's ratio to the aggregate modulus.

(2) $$H_{A}=\frac{1-\nu_{s}}{\left(1+\nu_{s}\right)\left(1-2\nu_{s}\right)}\;E_{\mathrm{qeq}}$$

### 2.5 Radiation Damage Assessment

The effects of radiation were assessed by mechanically testing cartilage samples before and after applying four different levels of radiation: 2, 48, 100, and 300 kGy, which are common total doses from previous synchrotron‐based measurements.^[^ ^68^, ^69^, ^70^ ^]^ Two kiloGray corresponds to a single 5 s scan, while 48 kGy corresponds to the maximal total dose in the current study, based on the unconfined compression StR. Absorbed dose was calculated by approximating AC samples as 4 mm diameter soft‐tissue cylinders (absorption coefficient µ/ρ = 0.7786 cm^2^ g^−1^ at 21 keV) following dose calculations applied in other biological systems in synchrotron‐based measurements.^[^ ^60^, ^70^, ^71^, ^72^ ^]^

Unconfined compression StR (1 step, 10% strain, speed of 1%/s, 10 min relaxation) was first applied ex‐beam to 3 AC samples per radiation group. The samples were then placed in the fridge (without freezing) to relax overnight. The following day, the samples were exposed to different radiation levels while rotating on the sample stage. Right after, the same mechanical protocol was applied again.

### 2.6 Histology

For comparative purposes, AC and MM tissue samples were fixed in 4% formaldehyde for 48 h, dehydrated in increasing ethanol concentration, embedded in paraffin, and sectioned to 5 µm thickness. Tissue sections were stained using hematoxylin and eosin to visualize tissue morphology, as well as Safranin O/fast green to visualize proteoglycan content. Histological slides were imaged using an Olympus BX43 light microscope.

### 2.7 Statistical Analysis

All results have been presented individually for each sample. No further statistical analysis has been performed due to the reduced sample size.

## 3 Results

### 3.1 Morphological Tissue Characterization

**Figure 2:**
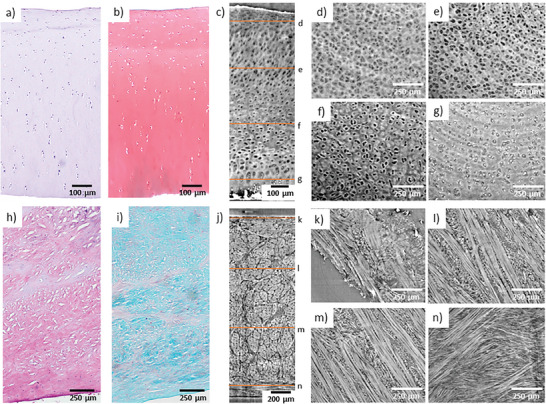
Morphological visualization in (a–g) articular cartilage (AC) and (h–n) medial meniscus (MM) samples, comparing histology and SR‐PhC‐µCT. Histological examples based on Hematoxylin and Eosin (a,h) and Safranin O/Fast Green (b,i) of the full tissue depth. c,j) Corresponding SR‐PhC‐ µCT images showing the full tissue depth, as well as cross‐sectional cuts that show (d–g) chondrocyte and (k–n) collagen fiber organization at different tissue depths. The SR‐PhC‐ µCT images are based on the defined protocol for “static” imaging.

Representative images from histology and SR‐PhC‐µCT from similar regions show the expected morphological features of AC and MM (**Figure** **2**). In AC, the main structural features that are visible in the SR‐PhC‐µCT images are chondrocytes. The chondrocytes are organized parallel and flatter at the surface and turn perpendicular, larger, and more organized into columns toward the deepest regions closer to the bone (Figure 2a–g). In MM, collagen fibers are more randomly orientated at the top and bottom layers while they form clear bundles of circumferentially oriented fibers in the middle layers (Figure 2h–n).

### 3.2 Image Quality Assessment

Image quality across varying scan times was assessed in terms of spatial resolution, SNR, and CNR. Visually, there was a very clear increase in image quality between 1 s and scans of 5 s or above, which is the reason why 5 s were chosen for dynamic imaging (Figure S2a–d, Supporting Information). The spatial resolution appeared rather constant between 3.3 and 3.5 pixels with a slight increase toward longer scan times, which could be an effect of image smoothing (Figure S2e, Supporting Information). SNR increased dramatically between 1 s and 5 s scans, to later plateau at scan times of 10 s or above (Figure S2f, Supporting Information). CNR followed a similar behavior as SNR especially for AC, while MM showed only a slight increase in CNR values from 1 s to the scans of 5 s or above (Figure S2g,h, Supporting Information).

### 3.3 Dynamic Investigation of Chondrocyte Distribution in Articular Cartilage

**Figure 3:**
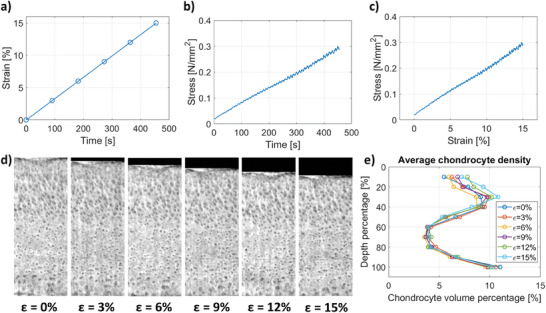
Chondrocyte density analysis in the cartilage under CL up to 15% strain. a) Strain‐time protocol applied. The data points indicate when a scan was acquired. Corresponding b) stress‐time and c) stress–strain response curves. d) Representative longitudinal SR‐PhC‐µCT slices for each strain level. e) Average chondrocyte density (chondrocyte volume percentage) per strain level across the cartilage depth (0% – surface). The SR‐PhC‐µCT images are based on the defined protocol for “dynamic” imaging.

**Figure 4:**
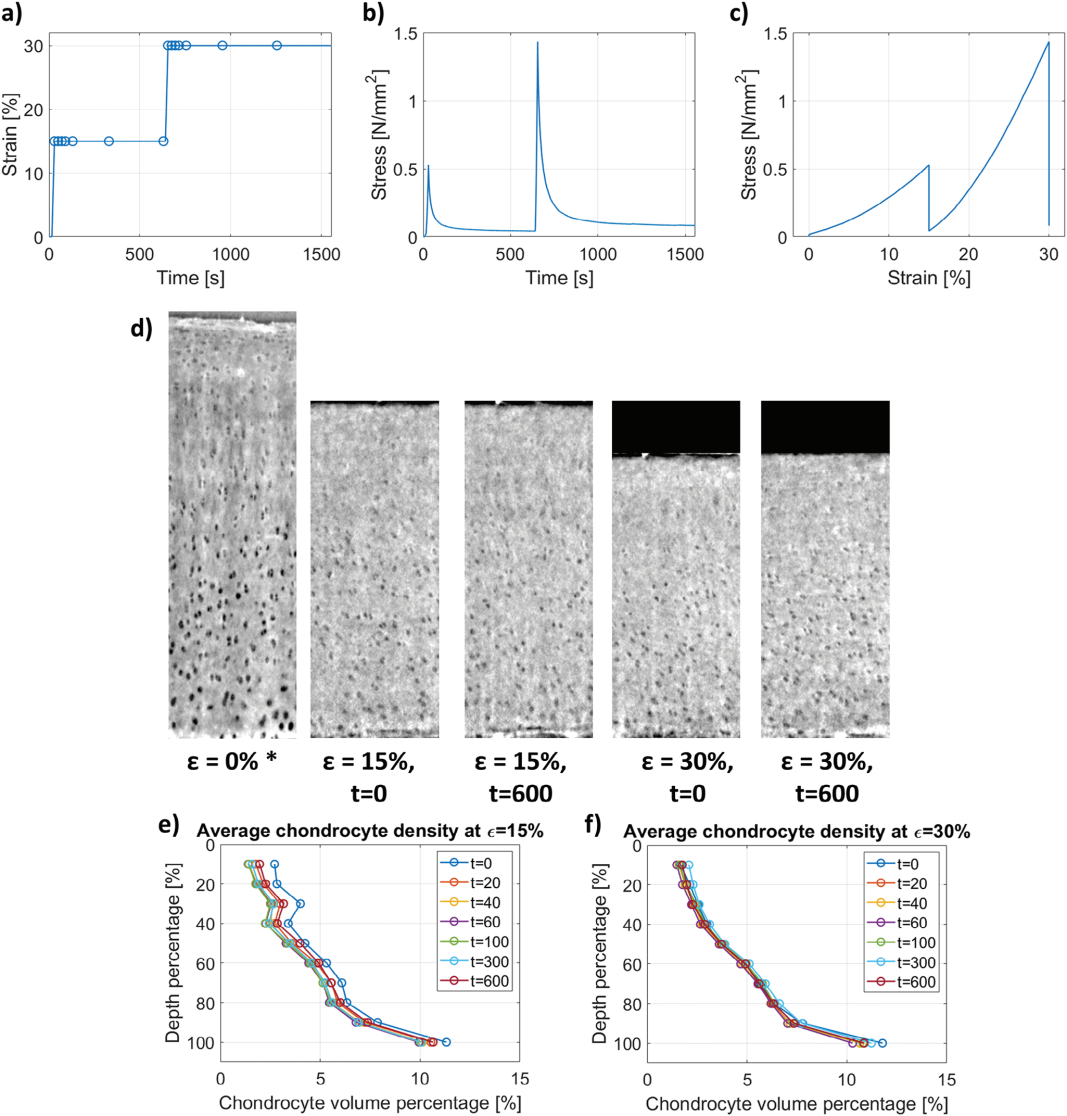
Chondrocyte density analysis in the cartilage under StR at 15% and 30% strain. a) Strain‐time protocol applied. The data points indicate when a scan was acquired. Corresponding b) stress‐time and c) stress–strain response curves. d) Representative longitudinal SR‐PhC‐µCT slices at 0%, 15% strain times 0 and 600, and at 30% strain times 0 and 600 (from left to right). *Indicates a similar static scan region, due to missing preload dynamic scan. e) Average chondrocyte density (chondrocyte volume percentage) across the cartilage depth (0% – surface) at f) 15% and g) 30% strain.

Simultaneous microstructural and mechanical response of AC samples was captured under CL and StR protocols (**Figures** **3**a–c and **4**a–c; Figure S3, Supporting Information). The resulting mechanical properties are summarized in Table S4 (Supporting Information).

During mechanical loading (both CL and StR protocols), chondrocyte density remained relatively constant, but slight differences were observed between CL (low strain rate) and StR (high strain rate). Under CL, most changes in chondrocyte density occurred in the top 40% of the cartilage depth while the rest of the tissue remained relatively constant during the mechanical test. This corresponds precisely with the change of orientation in the chondrocytes between the top and middle layers and was observed both in the images (Figure 3d) and from the quantitative analysis (Figure 3e). Under the first StR loading step, the chondrocyte density similarly varied the most in the superficial layer until ≈40% depth (Figure 4e). During the second StR loading step, when the sample was already compressed, the chondrocyte density remained rather constant during relaxation (Figure 4f).

### 3.4 Dynamic Investigation of Collagen Fiber Orientation in Meniscus

**Figure 5:**
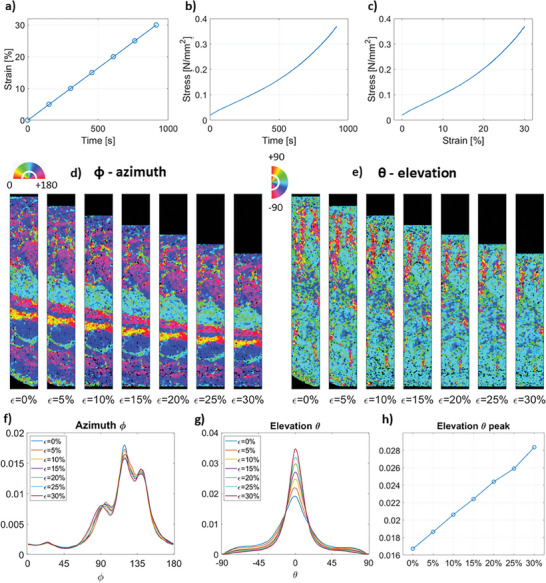
Collagen fiber orientation analysis in the meniscus under CL up to 30% strain. a) Strain‐time protocol applied. The data points indicate when a scan was acquired. Corresponding b) stress–time and c) stress–strain response curves. d) Azimuth and e) elevation angle maps of a representative longitudinal slice in a volume of interest across the acquired time points and corresponding strain values. f) Average azimuth and g) elevation angle histograms for all volumes of interest at each strain value. h) Evolution of the elevation angle peak height across acquired strain values. Vertical axes in (f,g,h) represent normalized counts.

**Figure 6:**
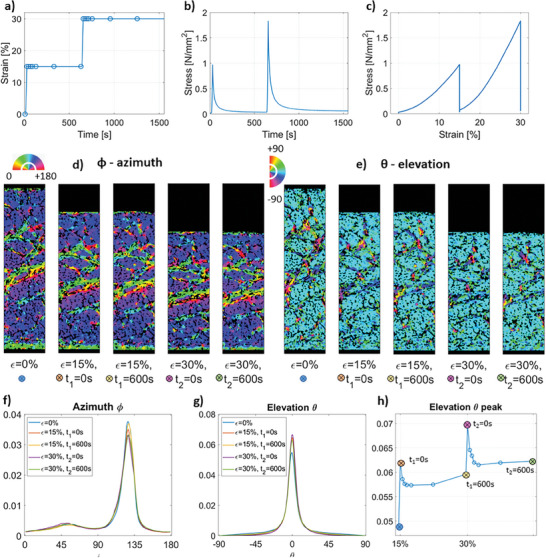
Collagen fiber orientation analysis in the meniscus under StR at 15% and 30% strain. a) Strain‐time protocol applied. The data points indicate when a scan was acquired. Corresponding b) stress–time and c) stress–strain response curves. d) Azimuth and e) elevation angle maps of a representative longitudinal slice in a volume of interest at preload, and first and last scans at 15% and 30% strain. f) Average azimuth and g) elevation angle histograms for all volumes of interest at each strain value. h) Evolution of the elevation angle peak height across acquired strain values. Color markers indicate the positions of each of the orientation maps in (d) and (e). Vertical axes in (f,g,h) represent normalized counts.

The simultaneous microstructural and mechanical behavior of meniscus samples was investigated under CL and StR protocols (**Figures** **5**a–c and **6**a–c). The resulting mechanical properties are summarized in Table S4 (Supporting Information).

Collagen fiber bundles were observed in all meniscus samples. These bundles were both characterized by either varying (layers with different orientations are shown as color variations in Figure 5d) or predominant azimuthal orientation across depth (as shown by the predominantly blue‐purple map in Figure 6d), which shows that different locations in the meniscus are organized following different orientation patterns. Most fiber bundles were found to be mainly oriented at 0 degrees of elevation angle (parallel to the surface, light blue), while some fibers were found organized following rather perpendicular orientation (Figures 5e and 6e).

During mechanical loading (both CL and StR protocols), the collagen fiber orientation remained relatively constant in the azimuth plane (Figures 5f and 6f) whereas fibers reoriented in the elevation plane, becoming more perpendicular to the loading direction (Figures 5g and 6g). In CL, the peak of the elevation angle histogram remained ≈0° but the corresponding amount of fibers at 0° increased linearly with the load (Figure 5g,h), which indicates that the amount of collagen fibers perpendicular to the loading direction increases with load and strain. In StR, the peak of the elevation angle histogram also remained ≈0°, and this time the amount of fibers at 0° followed a similar pattern to the stress–time curve (Figure 6g,h). That is, the number of collagen fibers at 0° peaked at maximal strain (t_1,2_ = 0s), and then quickly decreased during early relaxation (t_1,2_ < 100 s) to finally increase slightly toward the end of relaxation (100 s < t_1,2_ < 600 s).

### 3.5 Repeatability Analysis

Repeatability analysis of AC showed highly similar depth‐wise chondrocyte density distributions for the repeated scans of both exposure times (Figure S4, Supporting Information). The highest difference within the two repeated scans with the same exposure times was found in the deepest regions, where chondrocytes become larger than in the superficial layer and differences become more apparent. When comparing the 5 s dynamic and the 40 s static scans, the absolute difference value remained around or below 0.1% throughout the AC depth.

Repeatability analysis of MM showed almost identical azimuth and elevation maps for all acquisitions (Figure S5, Supporting Information). Corresponding histograms showed very similar distributions. Histogram differences remained very low for azimuth angle (<< 0.1%). In terms of elevation angle differences, these remained very low within the same scan time (<< 0.1%) but showed a noticeable peak height difference between scan times (≈0.2%).

### 3.6 Radiation Damage Assessment

There was no clear effect of radiation on the mechanical response of AC samples at 10% axial StR under different radiation regimes (2, 48, 100, and 300 kGy) (Figure S6, Supporting Information). Normal force differences pre‐ and post‐radiation remained around or below 1 N at the peak and went back to ≈0 N during most of the relaxation time. The estimation of mechanical properties from these curves showed small differences without any indication that these were caused by radiation damage, since the same property became higher or lower after irradiation, depending on the sample.

## 4 Discussion

This manuscript demonstrates the effectiveness of a customized rheometer setup for in situ loading simultaneously with synchrotron radiation‐based phase contrast imaging of biological non‐mineralized tissues. We focused on connective soft knee tissues, where we were able to capture the mechanical response of the tissues’ complex microstructural organization during different loading protocols, including continuous loading and stress relaxation. Thus, the strength of this setup hinges on the possibility of performing fast time‐resolved tomography of dynamic biological processes at high resolution and with high image quality.

Bovine AC and MM tissue samples have simultaneously been mechanically tested and imaged, while further analysis of the data showcased the possibilities offered by the presented methodology. The results have shown that image quality was sufficient to analyze the variation of chondrocyte distribution (Figures 3 and 4) and meniscus collagen fiber orientation (Figures 5 and 6). Moreover, through repeatability analysis and radiation damage tests, we provided insights about the variability in our quantitative results caused by repeated measurements, image processing, and mechanical behavior alteration from radiation. While our sample size was small, it is important to point out that the calculated mechanical properties were generally well within the reported range and variability in literature for unconfined compression with similar loading protocols.^[^ ^73^, ^74^, ^75^ ^]^

Compression of cartilage leads to complex changes within the tissue including matrix and chondrocyte deformation, hydrostatic and osmotic pressure, fluid flow, and altered water content.^[^ ^76^, ^77^, ^78^ ^]^ By tracking chondrocytes during compression, this study could directly visualize how articular cartilage loading is redistributed differently within zones showing that mostly in the top 40% of the tissue, chondrocyte density increases during compression. This is in good agreement with the fact that, when loaded, the complex hierarchical structure of articular cartilage results in a non‐uniform deformation field from the tissue scale down to the chondrocyte level.^[^ ^79^, ^80^, ^81^ ^]^ Furthermore, when cartilage is compressed, the chondron (chondrocytes and pericellular matrix together) deform differently in different zones.^[^ ^82^, ^83^ ^]^ Specifically, in the superficial zone, chondrons need more protection from the extracellular matrix strains in comparison to chondrons located deeper into the tissue where the strain magnitude is lower.^[^ ^82^, ^84^, ^85^ ^]^ This and other studies have shown how methodologies, such as the one here presented, that allow to study morphological and reorganizational changes of the chondrocytes during loading, may help in understanding structural alterations and biomechanical properties throughout the complex response of cartilage to mechanical stimulation.^[^ ^18^ ^]^

In the meniscus, our approach allowed us to identify fibers, fiber bundles, and larger regions of tissues that were characterized by the same fiber orientations, as well as to follow their behavior during loading. Circumferential fibers are characterized by substantial differences in their structural and mechanical properties depending on their location. In the inner zone, fiber density, and organization determine the mechanical response to a higher degree than proteoglycans and water content.^[^ ^86^, ^87^ ^]^ In our study, the samples were loaded axially in unconfined compression, which does not recapitulate the complexity of physiological loading. Additionally, using smaller tissue samples from one particular location within the joint does not address the variability of properties across the joint.^[^ ^88^ ^]^ Moreover, particularly for the meniscus, the use of smaller tissue samples may change the internal tissue response to mechanical compression. In an intact crescent‐shaped meniscus, the meniscal attachments enable transferring the axial load into the hoop (circumferential) stresses that are converted into tensile stresses along the collagen fibers in the meniscus.^[^ ^89^ ^]^ Thus, in the current study, we present proof of concept of a methodology that can identify and track alteration in the collagen fiber orientation inside meniscus tissue, albeit further adaptations are required before one can link it physiological response of the collagen fibers. In the future, our analysis could consider regions with different fiber orientations separately in order to compare their response to loading. Our study has also shown that during compression in our meniscus samples, the fibers reorient perpendicular to the loading direction, which then partially returns during relaxation. This may of course be specific to sample location, size, and loading configuration. Recently, other studies have investigated the nanostructural meniscal mechanical response by atomic force microscopy (AFM)‐nanoindentation^[^ ^86^, ^90^, ^91^ ^]^ and by in situ small angle X‐ray scattering^[^ ^15^ ^]^ detecting fibril rearrangement perpendicularly to the direction of the force, leading to a more oriented structure. An analogous behavior where fibrils and fibers aligned depending on the strain direction was also observed for stretched skin.^[^ ^92^, ^93^ ^]^ However, for these tissues fibrils and fibers reorient parallel to loading but still resulting in a more uniformly aligned fiber structure.^[^ ^86^ ^]^

In this study, we achieve a spatial resolution of ≈3 pixels (≈9 µm, Figure S2e, Supporting Information). In the meniscus, where the collagen type I fiber diameter ranges from 5 to 15 µm,^[^ ^94^ ^]^ it was possible to differentiate individual fibers and track their rearrangement during loading. However, in the case of AC, we primarily have type II collagen that does not form large fiber bundles like type I. In AC, fibril diameters vary from 40 to 60 nm,^[^ ^95^ ^]^ and therefore substantially below the resolution limit provided by the setup used in our experiment. Instead, the size of the chondrocytes (10–30 µm) provides clear contrast. Horng et al.^[^ ^18^ ^]^ have shown that it is possible to partially differentiate fibrils using nano‐holotomography under vacuum when reaching an effective voxel size of 0.1 × 0.1 × 0.1 µm^3^. However, this comes with the limitation of not being able to study hydrated samples (thus performing mechanical testing would not be feasible), and that the very small field of view would not allow imaging the whole depth of AC within one scan.

Variability in quantification was assessed through a repeatability analysis in which tissue samples were scanned twice at 40 s and twice at 5 s. The resulting data was then quantified separately and results were compared (Figures S4 and S5, Supporting Information). Variability in the range of 0.1–0.2% was observed for both chondrocyte segmentation and collagen fiber orientation histograms. The source of this variability is mainly the presence of noise, which can slightly alter the segmentation of features that are a few pixels wide (e.g., chondrocytes) in two different tomograms.

Cartilaginous tissues are composed of proteoglycans (≈1–3% for meniscus and 5–10% for cartilage), which are negatively charged sulfated glycosaminoglycan chains resulting in an osmotic swelling pressure. The swelling pressure helps maintain the structure and mechanical function of both the cartilage and meniscus.^[^ ^96^, ^97^ ^]^ In this study, we restricted the possible swelling of the samples by storing them in physiological saline and limiting the time in solution between thawing and testing. However, as the samples in our radiation damage test were stored in the fridge for 24 h, additional osmotic swelling could have influenced the mechanical properties (Figure S6b, Supporting Information).

The rheometer setup is highly versatile due to the possibility of adapting the sample environment with goal‐specific 3D‐printed geometries in combination with a range of loading protocols (e.g., compressive, tensile, axial and shear, among others) and time‐resolved imaging technology. While rheometers are commonly used in the lab for mechanical studies of soft musculoskeletal tissues,^[^ ^40^, ^98^, ^99^ ^]^ only one previous study combined their use with synchrotron‐based tomography.^[^ ^100^ ^]^ Kawano et al. studied the in situ deformation of AC using a rheometer and grating interferometry (GI).^[^ ^100^ ^]^ GI offers quantitative absorption, phase, and ultra‐small angle scattering contrast. While it can allow fast imaging for specific sample and imaging conditions,^[^ ^101^ ^]^ it is generally a slower imaging technique for matching resolution and image quality (it requires scanning a shifting grating for every tomographic projection and the beam is partially absorbed by the gratings) in comparison to the presented propagation‐based technique.^[^ ^102^ ^]^ Due to these limitations, deformation was studied between preload and relaxed state after compression. In the present study, this has been overcome by using a dedicated time‐resolved tomographic imaging setup and optimized scanning parameters, which allow to tackle soft‐tissue complexity both in terms of imaging and mechanical analysis. Therefore, the presented methodology opens the door to widely study the dynamic interplay between mechanics and microstructure not only in cartilage and meniscus, but in other biological non‐mineralized tissues of interest (e.g., tendon, muscle, vasculature wall, etc.) with better temporal resolution compared to what was previously achieved. In that way, both healthy and degenerated tissue states can be assessed in a reproducible manner to investigate the tissue microstructural properties as well as disease progression (e.g., osteoarthritis, healing, etc.).

When performing synchrotron experiments with biological tissues, careful planning is required to limit the radiation dose to avoid altering the tissue properties (e.g., collagen damage) as much as possible. The early study by Barth et al. set a reference level on mineralized bone tissue, stating that noticeable damage occurs after 35 kGy, while other studies showed that even high doses (230 kGy) do not necessarily alter all mechanical properties.^[^ ^103^ ^]^ In the present study, we kept the actual measurements at or below 48 kGy. However, we also tested radiation doses up to 300 kGy, without finding a significant impact on our measurements. Collagen damage/fragmentation may have still occurred on the molecular scale, but due to the tissue‐specific microstructural features and high fluid content, it did not adversely affect the measured mechanical response under the current loading protocols. However, radiation dose still needs to be considered and tested thoroughly when designing in situ experiments, as it can be highly variable for different tissues and dynamic measurements accumulate dose rapidly.

Future applications of the presented methodology to well‐selected cohorts of human tissue samples may provide a better understanding of the variability of structural parameters within donor specimens, as well as between donors of similar health status, possible gender effects, and moreover, how the microstructure is altered during tissue degeneration and progression of OA. With increasing degeneration, one would expect less dense and disorganized fibers, as well as a lower cell population and increased surface fibrillation.^[^ ^1^, ^2^, ^3^, ^104^ ^]^ Additionally, if both cartilage and meniscus from the same donors could be imaged, information regarding the timing of onset of early OA in each tissue could be determined using this approach. Moreover, data from recent in situ imaging studies of other musculoskeletal tissues and biomaterials have been combined with more advanced image analysis by, e.g., digital volume correlation (DVC).^[^ ^36^, ^37^, ^38^, ^105^, ^106^, ^107^, ^108^ ^]^ This would allow, in combination with the proposed methodology, the quantification of local strain distribution within tissues as well as tailoring the delivery of the data needed to validate novel computational models.^[^ ^109^, ^110^, ^111^ ^]^ However, this remains outside the scope of the current study.

### 4.1 Limitations

This proof‐of‐concept study presents some limitations. Currently, the setup requires the use of synchrotron sources to achieve the presented temporal resolution and image quality. Obtaining experimental time in synchrotron facilities is competitive, which limits access to this technology and therefore results in a small sample size. However, the setup has been highly optimized and thoroughly synchronized with the beamline, so that high throughput of samples can be achieved, although limited by the time required by each loading protocol (e.g., 10–15 min relaxation per load step in this study). Due to the small sample size, we refrain from drawing quantitative biological conclusions. Instead, we focus on presenting the extensive possibilities that this technique offers the community with the aim of presenting a novel methodology for the characterization of connective knee tissue, which can then be used for further and more complex biomedical studies. Currently, in situ SR‐PhC‐µCT is limited to a tissue sample size of a few millimeters, but future technical developments may allow the presented methodology to be applied to full knee structures or even joints. In addition, SR‐PhC‐µCT is currently limited to micrometer‐level structures with sufficient contrast. In the future, complementary scattering and diffraction measurements could provide further information on additional structures, such as collagen fibrils in AC. In terms of dynamic in situ measurements, one also needs to account for the deformation occurring during image acquisition, which can cause blurring and motion artefacts. This was tackled here by loading at slow speeds (CL) or uniquely during the relaxation time (StR). Finally, uncertainties and variations provided by image analysis and radiation damage are always important to assess, as done in our presented repeatability analysis and radiation damage analysis, showing little to no effect on our results.

## 5 Conclusion

This manuscript presented a novel methodology for the in situ mechanical and structural characterization of connective knee tissues. This setup enables the simultaneous dynamic investigation of microstructure and mechanics and thus has the potential to be extended to other loading protocols, a wide range of biological tissues, and medically relevant research questions.

## Conflict of Interest

The authors declare no conflict of interest.

## Supporting information

Supporting Information

## Data Availability

The data that support the findings of this study are available from the corresponding author upon reasonable request.
