# Supplementary material for: In Situ Loading and Time‐Resolved Synchrotron‐Based Phase Contrast Tomography for the Mechanical Investigation of Connective Knee Tissues: A Proof‐of‐Concept Study
Source: Adv Sci (Weinh). 2024 Mar 23;11(21):2308811. doi: 10.1002/advs.202308811 (PMC11151037; doi:10.1002/advs.202308811)
Supplement: Supplementary file 1 — Supporting Information [file ADVS-11-2308811-s001.pdf]

ADVANCED SCIENCE
Open Access

Supporting Information

for *Adv. Sci.*, DOI 10.1002/advs.202308811

In Situ Loading and Time-Resolved Synchrotron-Based Phase Contrast Tomography for the Mechanical Investigation of Connective Knee Tissues: A Proof-of-Concept Study

*Hector Dejea, Maria Pierantoni, Gustavo A. Orozco, E. Tobias B. Wrammerfors, Stefan J. Gstöhl, Christian M. Schlepütz and Hanna Isaksson**

Supplementary Material

In-situ Loading and Time-Resolved Synchrotron-Based Phase Contrast Tomography for the Mechanical Investigation of Connective Knee Tissues: a Proof-of-Concept Study

Hector Dejea, Maria Pierantoni, Gustavo A. Orozco, Edvin Tobias B. Wrammerfors, Stefan J. Gstöhl, Christian M. Schlepütz, Hanna Isaksson

Advanced Science, DOI:10.1002/advs.202308811 7

Table S1: Summary of sample numbers for each analysis. AC – articular cartilage; MM – medial meniscus; CL - continuous loading; StR – stress relaxation loading.

	**CL**	**StR**	**Image Quality**	**Repeatability Analysis**	**Radiation damage**
**AC**	1	1	1	1	12
**MM**	1	1	1	1	0

Table S2: Summary of the experimental parameters used in the static and dynamic synchrotron-based phase contrast tomography acquisitions.

	**Static / Dynamic**
**Energy**	21 keV
**Propagation distance**	40 cm
**Magnification**	4x
**Effective pixel size**	2.75 µm
**Field of view [mm^2]**	5.54 x 3.65 mm^2
**Field of view [pixels]**	2016 x 1400 pixels
**Projections**	4000 / 2000
**Darks**	50
**Flats**	400
**Exposure time**	9 ms / 2.5 ms
**Rotation angle**	180 degrees
**Time per scan**	40 s / 5 s
**Scintillator**	LuAG:Ce 150 µm
**Detector**	GigaFRoST

Table S3: Summary of the experimental parameters used to evaluate image quality between different scan times. Additional unchanged parameters can be found in Supplementary Table 1.

Total scan time	Exposure Time	Number of Projections
**1 s**	1 ms	1000
**5 s**	2.5 ms	2000
**10 s**	5 ms	2000
**20 s**	9 ms	2000
**40 s**	9 ms	4000

Table S4: Summary of the mechanical properties estimated from each mechanical test and sample. AC – articular cartilage; MM – medial meniscus; CL – continuous loading; StR – Stress-relaxation. For StR, the two numbers refer to the data from step 1 and step 2 respectively.

	E_i [MPa]	E_{qeq} [MPa]	Relaxation ratio [%]	Poisson ratio	H_A [MPa]
**AC – CL**	1.23	-	-	-	-
**AC – StR**	3.40, 9.28	0.26	8.27, 5.76	0.31, 0.23	0.33
**MM – CL**	1.19	-	-	-	-
**MM – StR**	6.36, 11.95	0.17	3.92, 3.48	0.26, 0.23	0.20

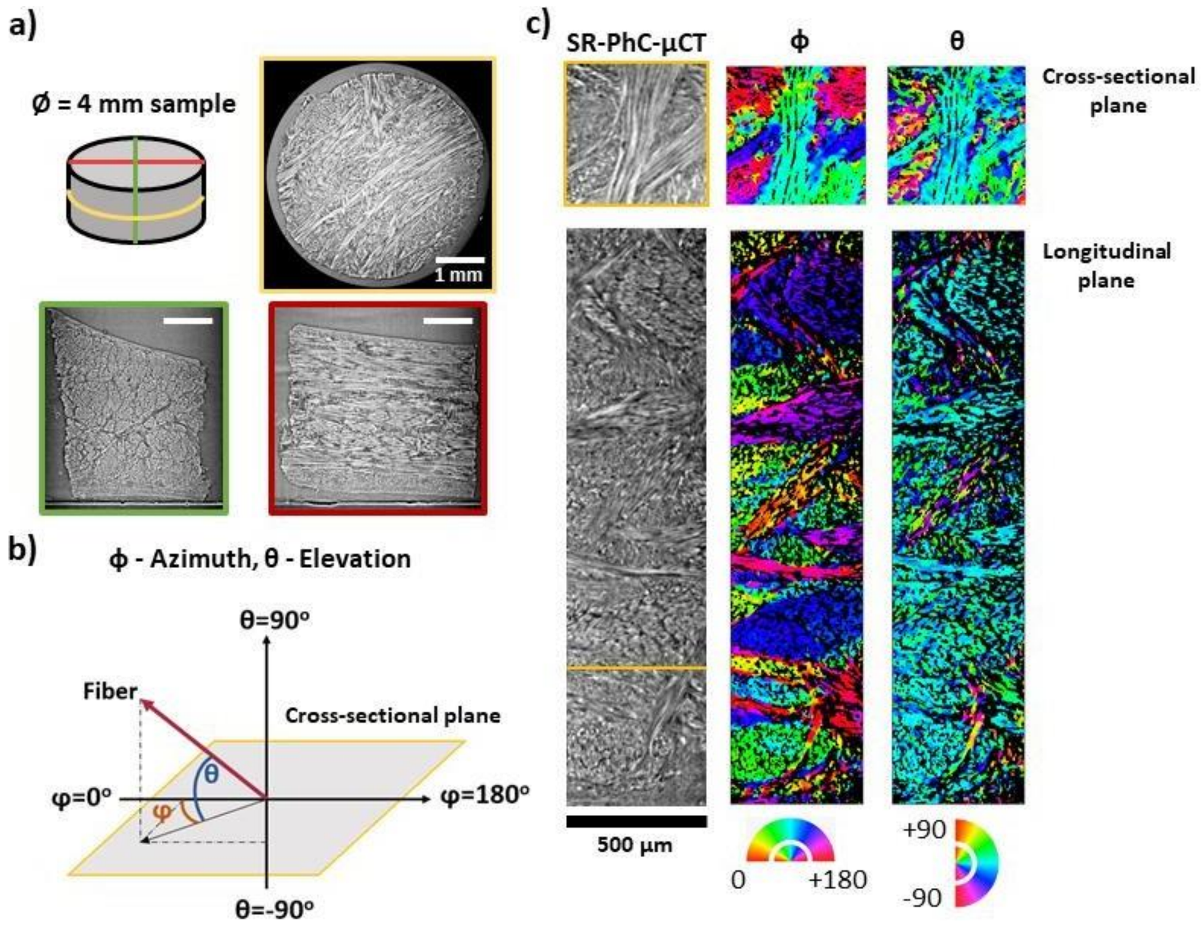

Figure S1: Demonstration of the structure tensor method application to calculate the orientation of meniscus collagen fibers. a) Diagram of a meniscus tissue sample and illustrative orthogonal slices. Scale bar is 1 mm. b) Sketch indicating the azimuth and elevation angles of an example fiber. b) Illustrative SR-PhC-µCT with representative ROI for analysis in yellow. c) Illustrative ROI cross-sectional and longitudinal plane images corresponding to SR-PhC-µCT slices, azimuth angle and elevation angle (from left to right).

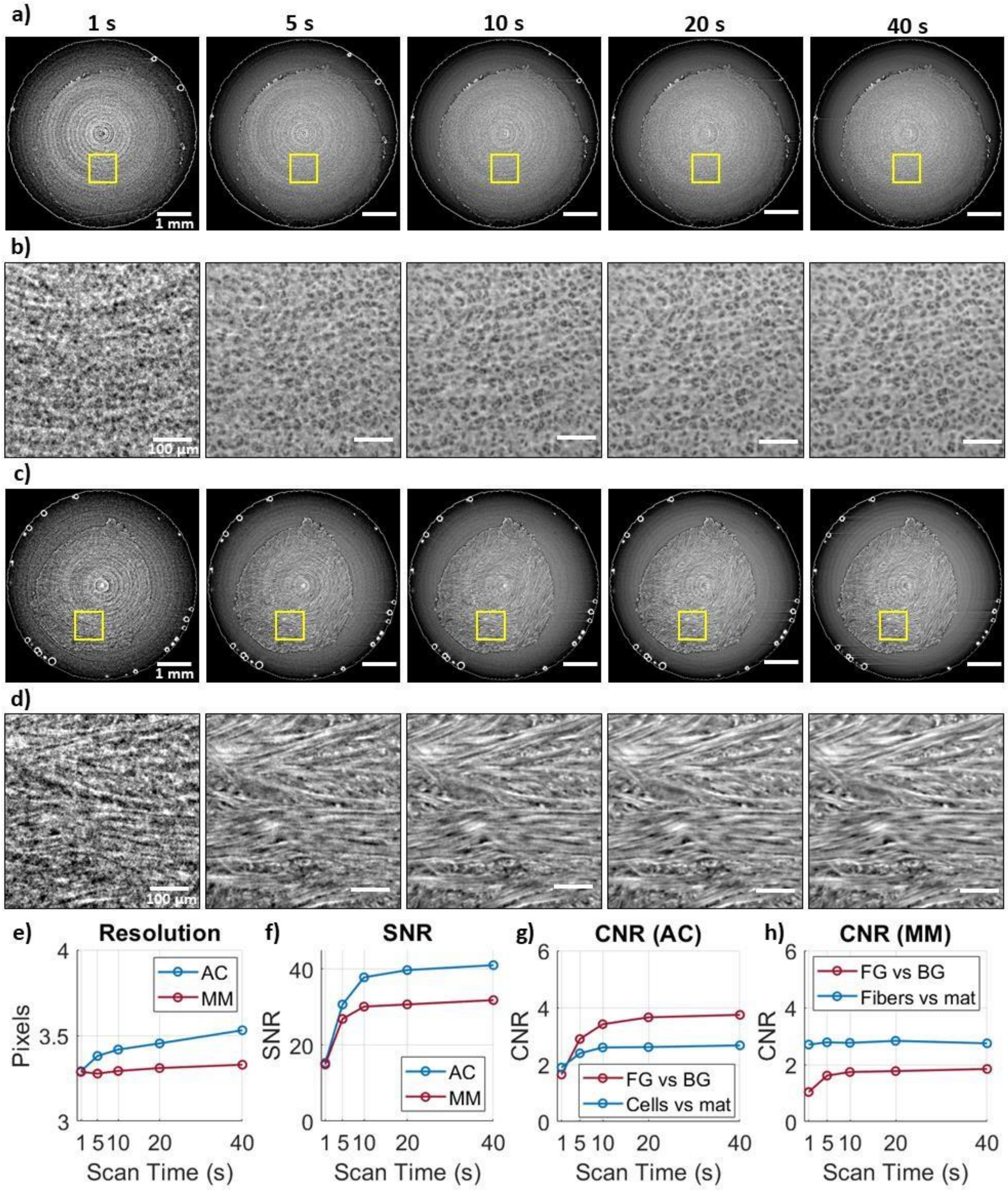

Figure S2: Image quality analysis at varying scan times. a) Equivalent representative slice of an articular cartilage (AC) sample at scan times of 1 s, 5 s, 10 s, 20 s and 40 s, from left to right respectively. b) Zoom in of the yellow region of interest marked in a). c) Equivalent representative slice of a medial meniscus (MM) sample at scan times of 1 s, 5 s, 10 s, 20 s and 40 s, from left to right respectively. d) Zoom in of the yellow region of interest marked in c). Image quality characterization plots in terms of e) resolution, f) signal-to-noise ratio (SNR), contrast-to-noise ratio (CNR) for g) foreground vs background (FG vs BG) and cells vs matrix in AC, and h) for FG vs BG and fibers vs matrix in MM. Scale bar is 1 mm in a) and c), and 100 µm in b) and d).

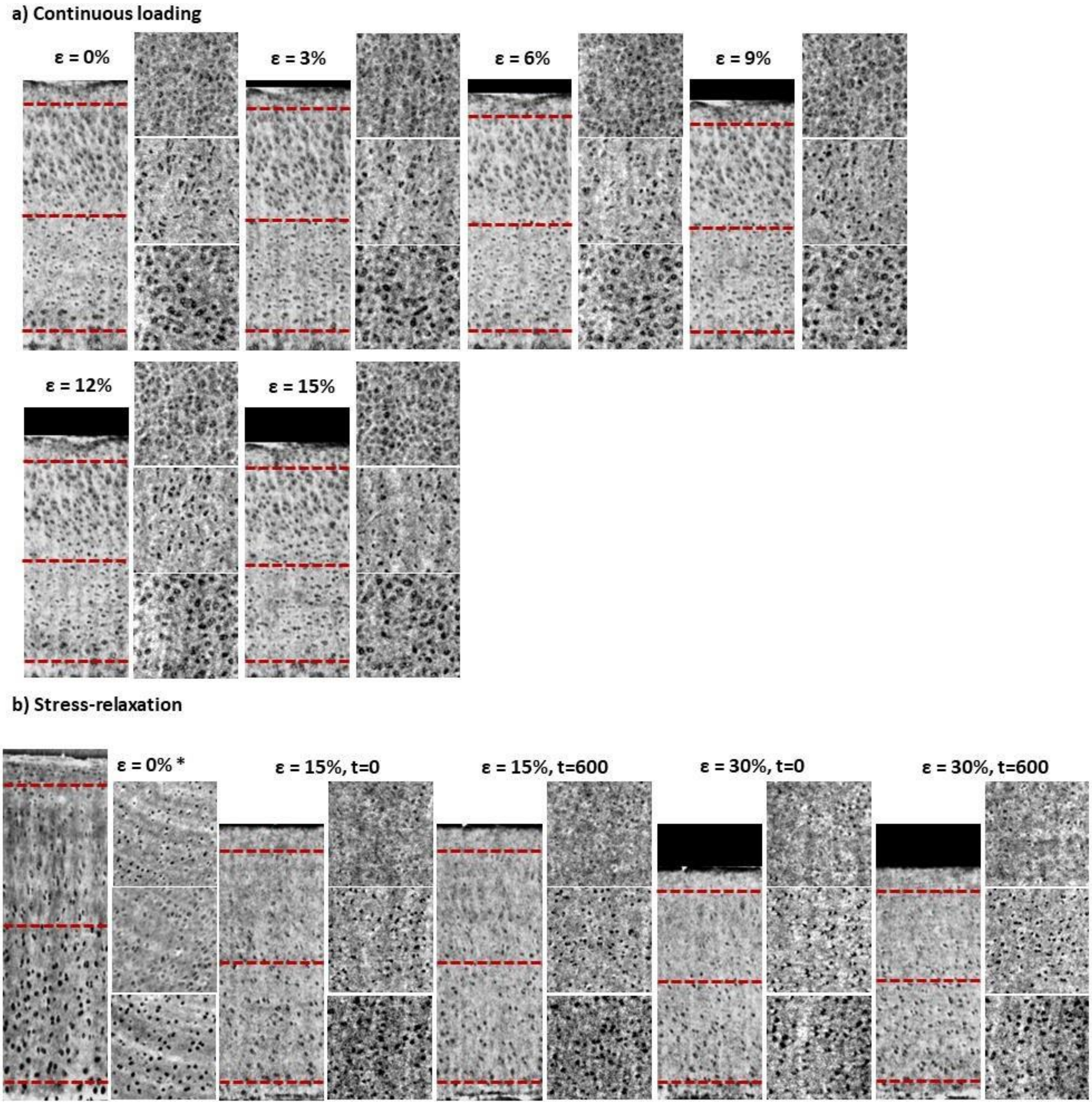

Figure S3: Longitudinal and corresponding cross-sectional cuts for different strain and relaxation steps in a) continuous loading and b) stress-relaxation protocols for articular cartilage samples. *Indicates a similar static scan region, due to missing preload dynamic scan. All images have a width of 500 µm.

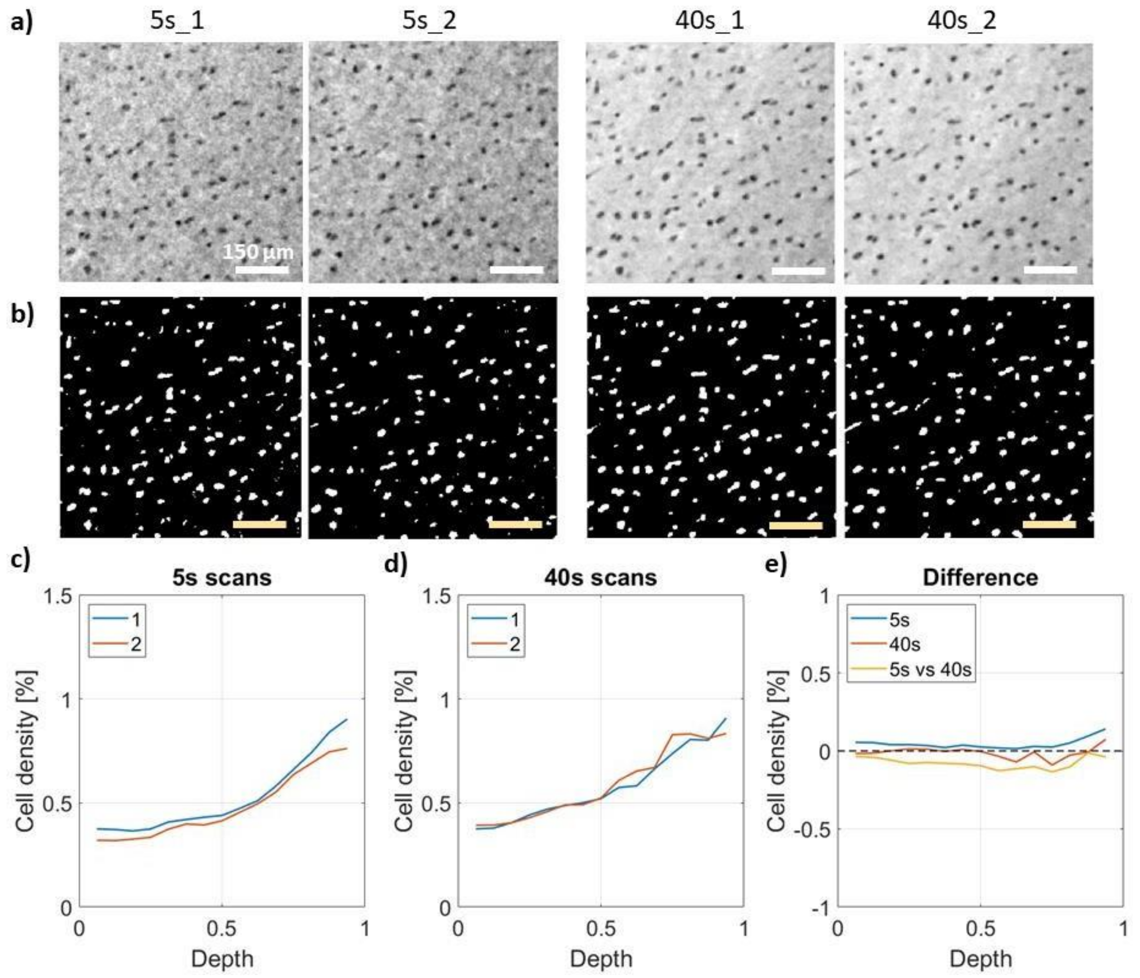

Figure S4: Repeatability analysis of chondrocyte segmentation in cartilage samples. a) Representative tomographic reconstruction cross-sectional slices of the 5s scans 1 and 2, and the 40s scans 1 and 2 (from left to right). b) Corresponding chondrocyte segmentation of the slices in a). c) Depth-wise chondrocyte density for 5s and d) 40s scans. e) Difference in depth-wise chondrocyte density between 5s scans, 40s scans, and the average 5s and 40s curves. Scale bar is 150 µm.

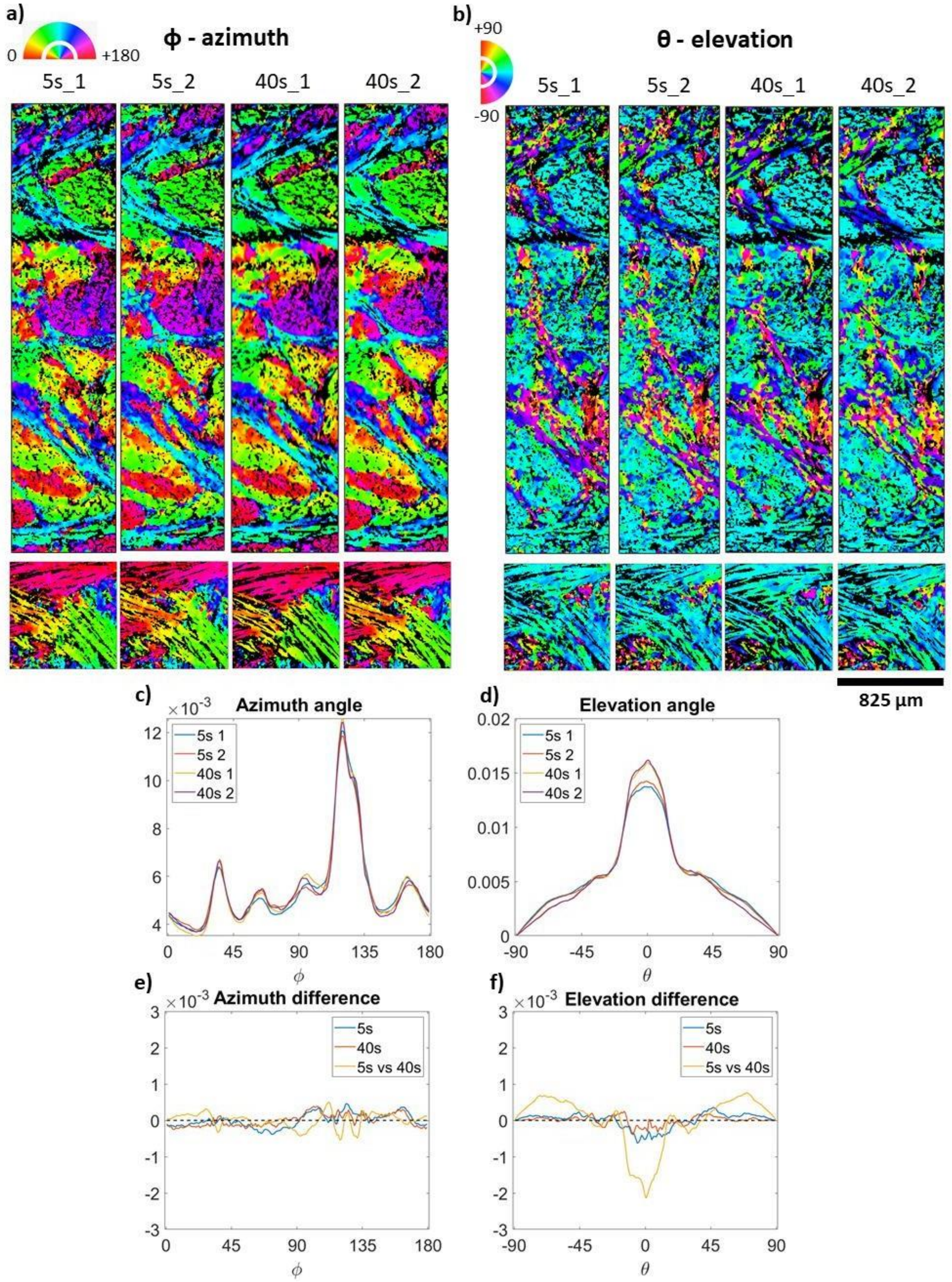

Figure S5: Repeatability analysis of collagen fiber orientation in meniscus samples. a) Azimuth angle results in representative longitudinal and cross-sectional slices for 5s scans 1 and 2, and 40s scans 1 and 2 (from left to right). b) Elevation angle results in the same representative slices shown in a). c) Azimuth and d) elevation angle histograms for each scan. e) Differences in azimuth and f) elevation angle histograms between 5s scans, 40s scans, and between the average 5s and 40s histograms.

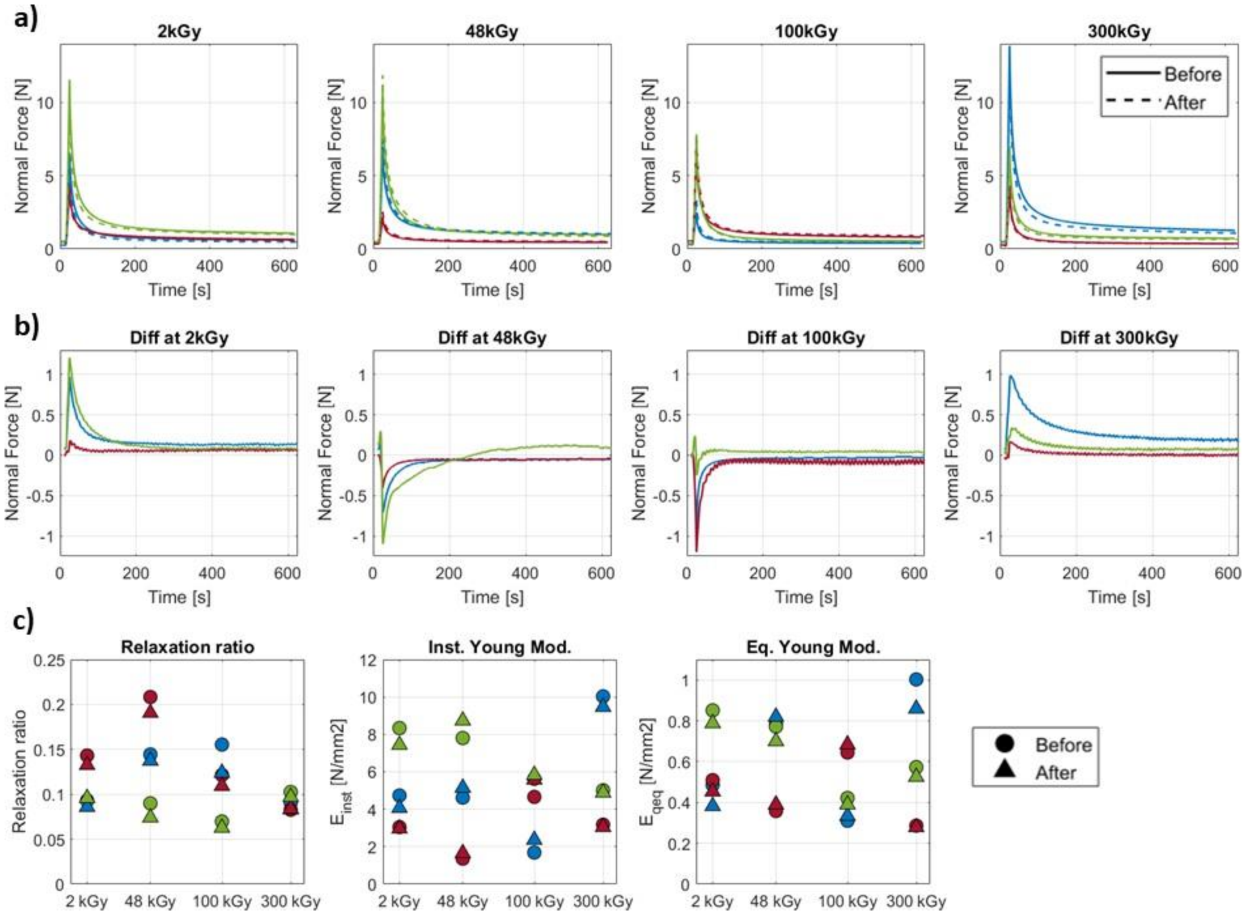

Figure S6: Radiation damage analysis based on the mechanical properties of articular cartilage (AC) samples. a) Normal force measurements of 3 tissue samples before (bold line) and after (dotted line) applying 2 kGy, 48 kGy, 100 kGy and 300 kGy (from left to right). Same color indicates same sample. b) Difference in normal force between pre- and post-radiation measurements. c) From left to right, relaxation ratios, instantaneous Young's modulus and equilibrium Young's modulus before (circles) and after (triangles) applying the different levels of radiation. Same color indicates same sample.

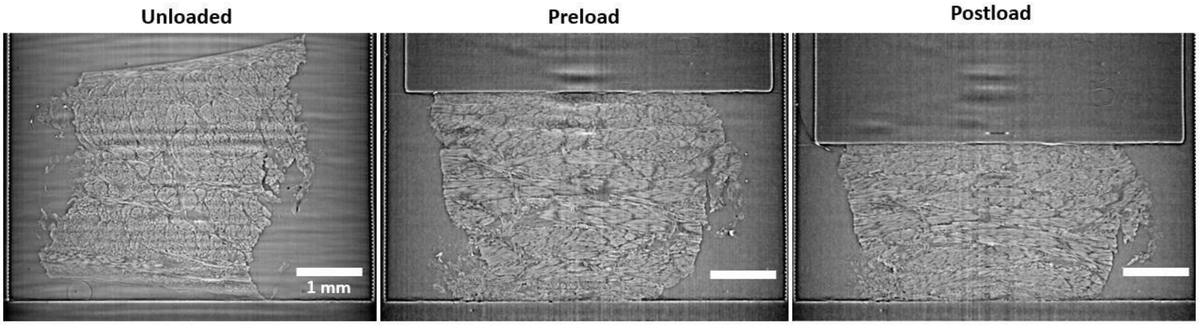

Figure S7: Example slices of unloaded, preload and postload states for a meniscus tissue sample. Scale bar is 1 mm.
